# Hyperdirect projections from the supplementary motor area mediate subthalamic stimulation effects on movement initiation and inhibition in Parkinson’s disease

**DOI:** 10.1093/braincomms/fcag325

**Published:** 2026-08-28

**Authors:** Garance M Meyer, Marion Albares, Guillaume Lio, Alina Beliakova, Gustavo Polo, Brian Lau, Andreas Horn, Stéphane Thobois, Bénédicte Ballanger, Philippe Boulinguez

**Affiliations:** Université Lyon 1, CNRS, INSERM, Centre de Recherche en Neurosciences de Lyon CRNL, UMR 5292, UMR S1028, Bron F-69500, France; Harvard Medical School, Boston, MA 02138, USA; Brigham and Women’s Hospital, Department of Neurology, Center for Brain Circuit Therapeutics, Bos, MA 02115, USA; Université Lyon 1, CNRS, INSERM, Centre de Recherche en Neurosciences de Lyon CRNL, UMR 5292, UMR S1028, Bron F-69500, France; iMIND, Center of Excellence for Autism, le Vinatier Hospital, Bron F-69500, France; CNRS, UMR 5229, Institute of Cognitive Science Marc Jeannerod, Bron F-69500, France; Université Lyon 1, CNRS, INSERM, Centre de Recherche en Neurosciences de Lyon CRNL, UMR 5292, UMR S1028, Bron F-69500, France; Hospices Civils de Lyon, Hôpital Neurologique Pierre Wertheimer, Neurochirurgie A, Bron F-69500, France; Sorbonne Université, Institut du Cerveau—Paris Brain Institute—ICM, Inserm, CNRS, APHP, Hôpital de la Pitié Salpêtrière, Paris 75013, France; Harvard Medical School, Boston, MA 02138, USA; Brigham and Women’s Hospital, Department of Neurology, Center for Brain Circuit Therapeutics, Bos, MA 02115, USA; Network Stimulation Institute, Department of Stereotactic and Functional Neurosurgery, University Hospital Cologne 50937, Germany; Université Lyon 1, CNRS, INSERM, Centre de Recherche en Neurosciences de Lyon CRNL, UMR 5292, UMR S1028, Bron F-69500, France; Hospices Civils de Lyon, Hôpital Neurologique Pierre Wertheimer, Neurologie C, Centre Expert Parkinson, Bron F-69500, France; Université Lyon 1, CNRS, INSERM, Centre de Recherche en Neurosciences de Lyon CRNL, UMR 5292, UMR S1028, Bron F-69500, France; Université Lyon 1, CNRS, INSERM, Centre de Recherche en Neurosciences de Lyon CRNL, UMR 5292, UMR S1028, Bron F-69500, France

**Keywords:** Parkinson’s disease, deep brain stimulation, subthalamic nucleus, response inhibition, impulsivity

## Abstract

In Parkinson’s disease, subthalamic deep brain stimulation improves movement initiation but can induce impulsivity. The origin of these effects remains unclear. Here, we test the hypothesis that modulation of automatic response inhibition, an underrecognized process that governs the ability to refrain from reacting, accounts for both effects. Nineteen Parkinson’s disease patients were assessed for (i) behavioural changes in a simple Go/NoGo task, ON versus OFF deep brain stimulation, (ii) structural connectivity between the stimulated subthalamic region and the supplementary motor complex and (iii) a previously established electroencephalographic marker of automatic response inhibition (the DMF170). Under stimulation, patients exhibited faster reaction times and increased commission errors. These changes were accompanied by an increase in DMF170 amplitude, which correlated with reaction times. Finally, behavioural changes were associated with stimulation volume overlap with fibres connecting the subthalamic nucleus to the supplementary motor complex. Our findings link improved movement initiation and increased impulsivity under stimulation of the subthalamic nucleus to a shared anatomical and functional substrate, namely the subthalamic nucleus-supplementary motor cortex loop supporting automatic response inhibition.

## Teaser

In Parkinson's disease, subthalamic deep brain stimulation improves movement, but can increase impulsive actions. By studying patients on and off stimulation, Meyer *et al*. show that these effects arise from changes in response inhibition, supported by a shared neural pathway linking the subthalamic nucleus and supplementary motor cortex.

## Graphical Abstract

**Graphical Abstract:**
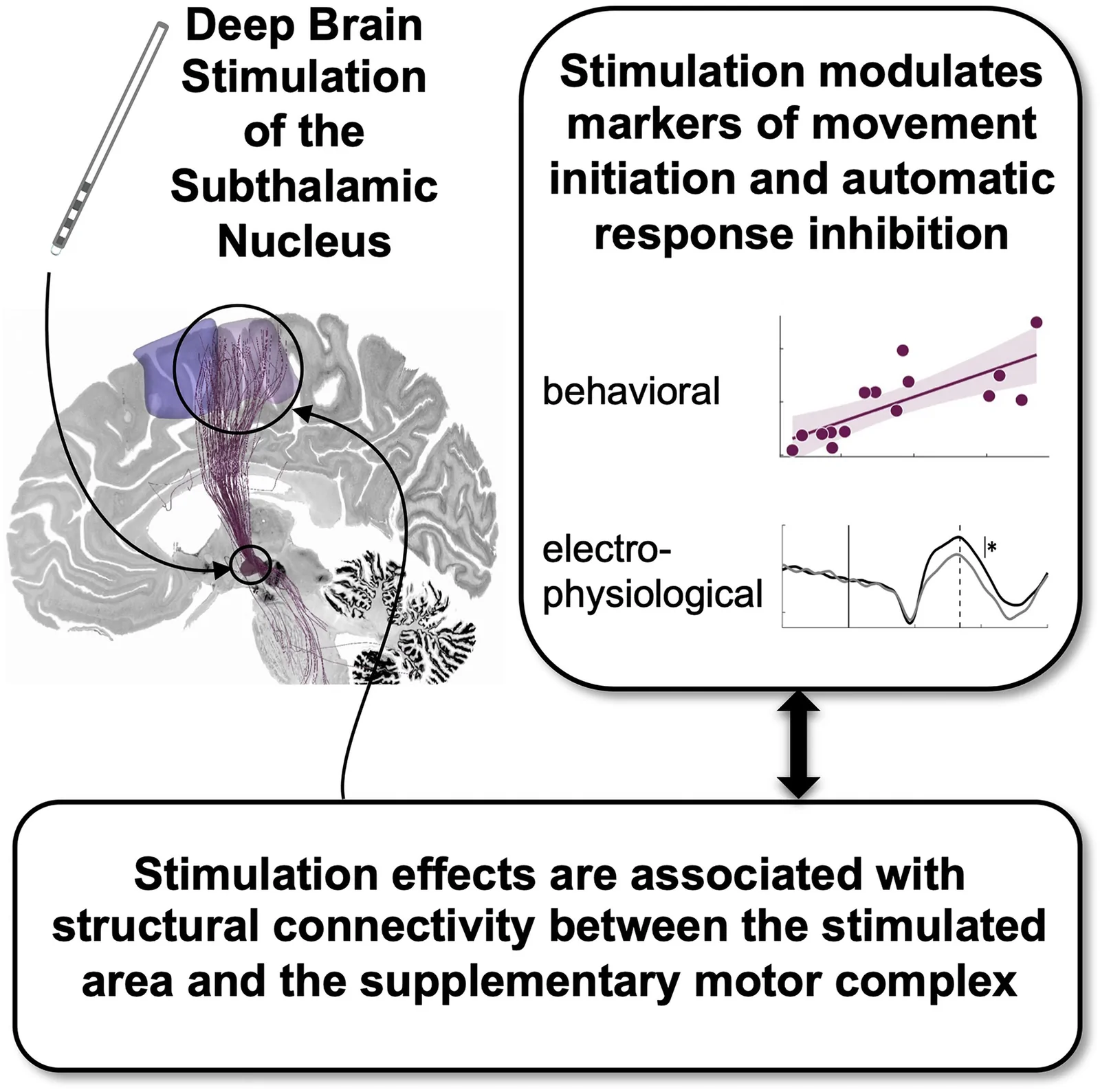

## Introduction

Deep brain stimulation of the subthalamic nucleus (STN-DBS) is an established and increasingly used therapeutic option for Parkinson’s disease.^1-3^ While STN-DBS effectively relieves motor symptoms, including movement initiation dysfunctions that can be difficult to treat using dopaminergic medication,^4^ it can also induce side-effects such as inhibitory dysfunctions, namely action impulsivity.^5-9^ Overall, STN-DBS outcomes are variable and rely on numerous factors, some of which are unrelated to the stimulation itself.^10-12^ Still, it is crucial to characterize the neural mechanisms and brain circuits underlying the therapeutic and adverse effects of DBS to improve outcomes.^13,14^

Several limitations impede our understanding of the effects of STN-DBS on movement initiation, such that it remains unclear which executive or sensorimotor mechanisms and associated networks are modulated by stimulation.^4-6,15-17^ First, predicting the extent of the affected networks is difficult because it requires precise estimation of the STN area affected by stimulation. Indeed, the STN is organized along a gradient between dorsolateral motor, intermediate associative and ventromedial limbic territories, which are embedded into distinct functional networks.^8,18,19^ The hyperdirect pathway, in particular, is an important pathway by which the cortex directly modulates basal ganglia activity via the STN,^20-22^ and likely mediates some of the therapeutic and adverse effects of DBS,^23-25^ but it remains difficult to associate its different subsections to specific functions and symptoms.^18,19^ Second, our ability to assess DBS network effects has been historically limited by the difficulty in performing whole brain activity recordings under DBS using fMRI, MEG or EEG due to safety concerns or artefacts.^26-28^ Third, the control of movement initiation relies on response inhibition, a function consisting of a variety of processes that are still debated in healthy subjects,^29-34^ whose dysfunctions are also conflicting in PD patients,^4,35,36^ and that might be differentially modulated by STN-DBS.^5,6,37^

Action impulsivity and disinhibition have mostly been associated with the impairment of the ability to *stop ongoing movements*,^38-40^ a process which consists in identifying the stimulus that explicitly instructs stopping, and then generating an online inhibitory command to cancel the movement before it has been fully completed.^29,41^ This relies on the section of the hyperdirect pathway connecting the inferior frontal gyrus (IFG) and STN.^29,39,42-47^ However, STN-DBS effects on stopping have not been associated with the beneficial effects of stimulation on movement initiation.^39,48^ We propose that the modulation of automatic response inhibition, a different but complementary inhibitory process that sets the ability to *refrain from reacting,* might account for both improvement of movement initiation and appearance of action impulsivity under STN-DBS. This function, which has been far less investigated to date, consists of the automatic inhibition of any fast motor response before its initiation as soon as the context is uncertain (i.e. when more time is required to fully identify the stimulus and decide on the best course of action;^9,32,49-52^). It likely relies on different hyperdirect projections connecting the pre-SMA/SMA complex (SMC) and STN.^32,33,51^ Specifically, this function might play a major role both in movement initiation dysfunctions observed in PD at the pathological state, and in beneficial as well as adverse effects of STN-DBS.^4,9,35,36,53^ Indeed, difficulties in setting an appropriate level of automatic inhibition according to the context might lead to difficulties in initiating movements (akinesia; excessive automatic inhibition) or, conversely, to difficulties in refraining from reacting to external stimuli (impulsivity; lack of automatic inhibition).

To test our hypothesis, we implemented a simple Go/NoGo task eliciting action restraint^32,51^ in both ON- and OFF-DBS states in 19 patients with PD. We used high-density EEG and source-space analyses to track an electrophysiological signature of automatic response inhibition in the supplementary motor cortex. Finally, we modelled the stimulation volumes and identified the structural connections whose stimulation may account for DBS effects.

## Materials and methods

### Patients

Nineteen patients with idiopathic PD and bilateral STN-DBS (5 females, 14 males, 59.7 ± 7.2 years old, disease duration: 13.4 ± 5.9 years, Levodopa equivalent daily dose: 547.0 ± 274.6) were tested while taking their regular antiparkinsonian medication on two consecutive days in ON- and OFF-DBS states, in a randomized order. Testing included the UPDRS-III and the behavioural task. For the OFF state, stimulation was turned off 35 min before starting. For the ON state, clinical stimulation settings were used. Detailed information on patients, including clinical characteristics and stimulation parameters, is available as Supplementary Material 1.

Patients were included if they had received STN-DBS for at least 3 months and had a stable dopaminergic treatment for at least 2 months. Exclusion criteria were dementia (MATTIS < 130), presence of another neurological or psychiatric disease (except depression), substance use or dependence according to the DSM-IV-TR criteria (except tobacco smoking), being treated for another medical condition with a medication that would have psychological or neurological effects. The patients took part in the experiment during their regular follow-up visits and were compensated 120€ for their participation in the entire study, which included two other experimental sessions. All patients provided written informed consent. The research was conducted in accordance with the Declaration of Helsinki and approved by the local ethics committee (CPP sud-est IV, N°11/097).

### Electrode localization and estimation of stimulation volumes

We used the Lead-DBS toolbox (v2.5, www.lead-dbs.org) to localize the DBS electrodes (model 3389, Medtronic) in template ‘MNI’ space based on the preoperative and postoperative imaging, as described elsewhere.^54,55^ For this analysis only, four patients were excluded because the imaging was unavailable (*N* = 3) or because of extensive atrophy precluding accurate normalization (*N* = 1), leading to a sample size of *N* = 15. All patients (*N* = 19) were included in the behavioural and EEG analyses.

Briefly, we used the standard Lead-DBS analysis pipeline, which includes linear co-registration of the pre- and post-operative images, normalization into MNI template space, brain shift correction and electrode reconstruction.^54,55^ At each step of the pipeline, the results were carefully checked by visual inspection using the built-in tools. In particular, the accuracy of the normalization is critical for correct electrode localization and was verified with special attention to the correspondence of template and normalized patient images at published subcortical anatomical fiducials.^56^ When necessary, manual corrections of the normalization and electrode reconstruction were applied using the dedicated tools in Lead-DBS.^54,57^

The stimulation volumes (so-called ‘volumes of tissue activated’, or VTAs;^58,59^) were estimated based on electric field (E-field) modelling using the ‘Simbio/Fieldtrip’ method implemented in Lead-DBS.^54,55,60^ This method estimates the E-field distribution in a four-compartment tetrahedral mesh (electrode material, contacts and white and grey matter as defined based on the DISTAL atlas;^61^) based on a finite element model. Preset values of 0.14 S/m and 0.33 S/m were used for the conductivity of white and grey matter, respectively. Finally, the binary stimulation volumes were obtained by thresholding the electric field magnitude maps at a heuristic value of 0.2 V/mm.^59^ Of note, all patients received monopolar stimulation.^62^ Active contact locations and distribution of the stimulation volumes across patients are shown in Fig. 1.

**Figure 1:**
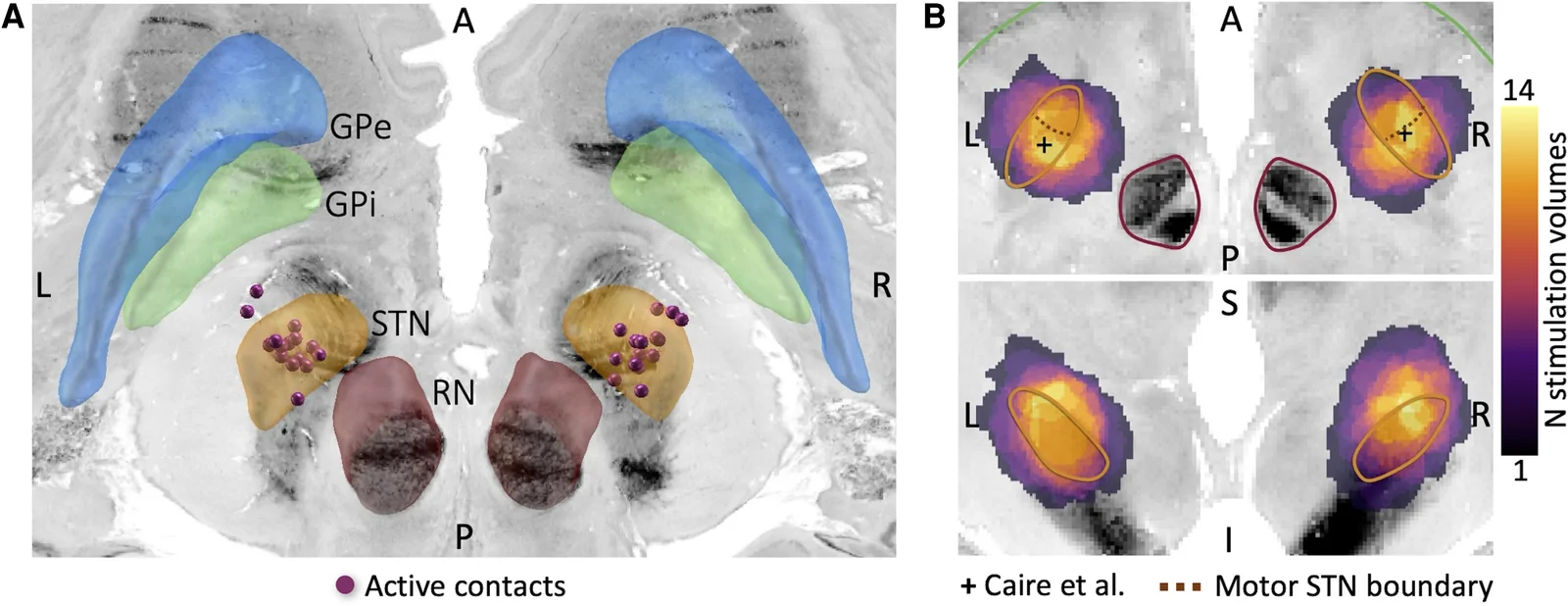
**Active contact locations (A) and overlap of stimulation volumes (‘N-map’; (B) axial and coronal views) for the 15 patients included in structural connectivity analyses.** Most active contacts were clustered in and close to the subthalamic nucleus, and strongest overlap between the stimulation volumes (i.e. E-field magnitude thresholded and binarized at >0.2 V/mm;^59^) across patients was found in the dorsolateral, motor aspect of the subthalamic nucleus, in the vicinity of the optimal stimulation site (‘sweet-spot’) coordinates reported by Caire *et al*.^63^; indicated by a cross on the axial view. Indeed, the stimulation volumes encompassed the motor subthalamic nucleus as defined by the DISTAL atlas (^61^; delimited by the dashed lines on axial view; coronal view taken within the motor subthalamic nucleus) in all patients and hemispheres, albeit to varying extents. Of note, the surrounding white matter may also contribute to clinical benefit.^64,65^ GPe: globus pallidus pars externa; GPi: globus pallidus pars interna; STN: subthalamic nucleus; RN: red nucleus.

### Structural connectivity between the stimulated volume of the subthalamic nucleus and the supplementary motor complex

We used the DBS fiber-filtering method (fully described in^54^) to identify fibre tracts whose intersection with stimulation volumes was associated with stronger behavioural effects. This was based on a PD-specific normative connectome (‘PPMI-85’) derived from high-quality diffusion-weighted imaging of 85 PD patients from the Parkinson’s Disease Progression Marker Initiative (^61,66^; and openly available in Lead-DBS). Following on recent publications, the analysis used unthresholded E-fields rather than binary stimulation volumes.^67,68^ Only the fibres traversing at least 20% of the E-fields above a threshold of 0.2 V/mm were considered (which corresponds to default settings). Each of these fibres was assigned a value representing the Spearman coefficient of correlation -calculated across patients- between the ‘impact’ of each patient’s E-fields on the fibre (i.e. peak E-field magnitude along the fibre) and behavioural change. The 150 top fibres were retained (corresponding to 0.62 < *ρ <* 0.84 for commission error rate and 0.53 < *ρ <* 0.90 for RT). The Pearson correlation coefficient between the patients’ ‘fiber scores’ (representing the weighted sum of the fibre values traversing a patient’s E-fields) and the behavioural variable is reported as a summary statistic. Of note, because the ‘fiber scores’ are based on E-fields, this metric indirectly accounts for stimulation amplitude and contact configuration. Leave-one-out cross-validation was performed to assess the robustness of the results.^68^ To pinpoint the key cortical regions in the identified (structural) network, follow-up analyses were run to test for correlation between behavioural variables and structural connectivity of the stimulation volumes with specific regions of interest (SMA, pre-SMA, IFG, primary motor cortex from the AAL3 atlas).^69^

### Behavioural experiment and EEG recordings

We used a simple, refined Go/NoGo task eliciting automatic inhibition of prepotent responses (fully described elsewhere^32,51^; Fig. 2A). Patients were asked to respond as fast as possible with a right thumb button press to Go signals (green diamonds), while refraining from responding to NoGo signals (green crosses). Two conditions were presented in separate blocks, with equiprobable Go and NoGo trials in the mixed condition (uncertain context, requiring action restraint), and only Go trials in the control condition (fully predictable context, allowing automatic responses). The behavioural variables of interest were Go reaction times (RT) and commission errors (responses to the NoGo).

**Figure 2:**
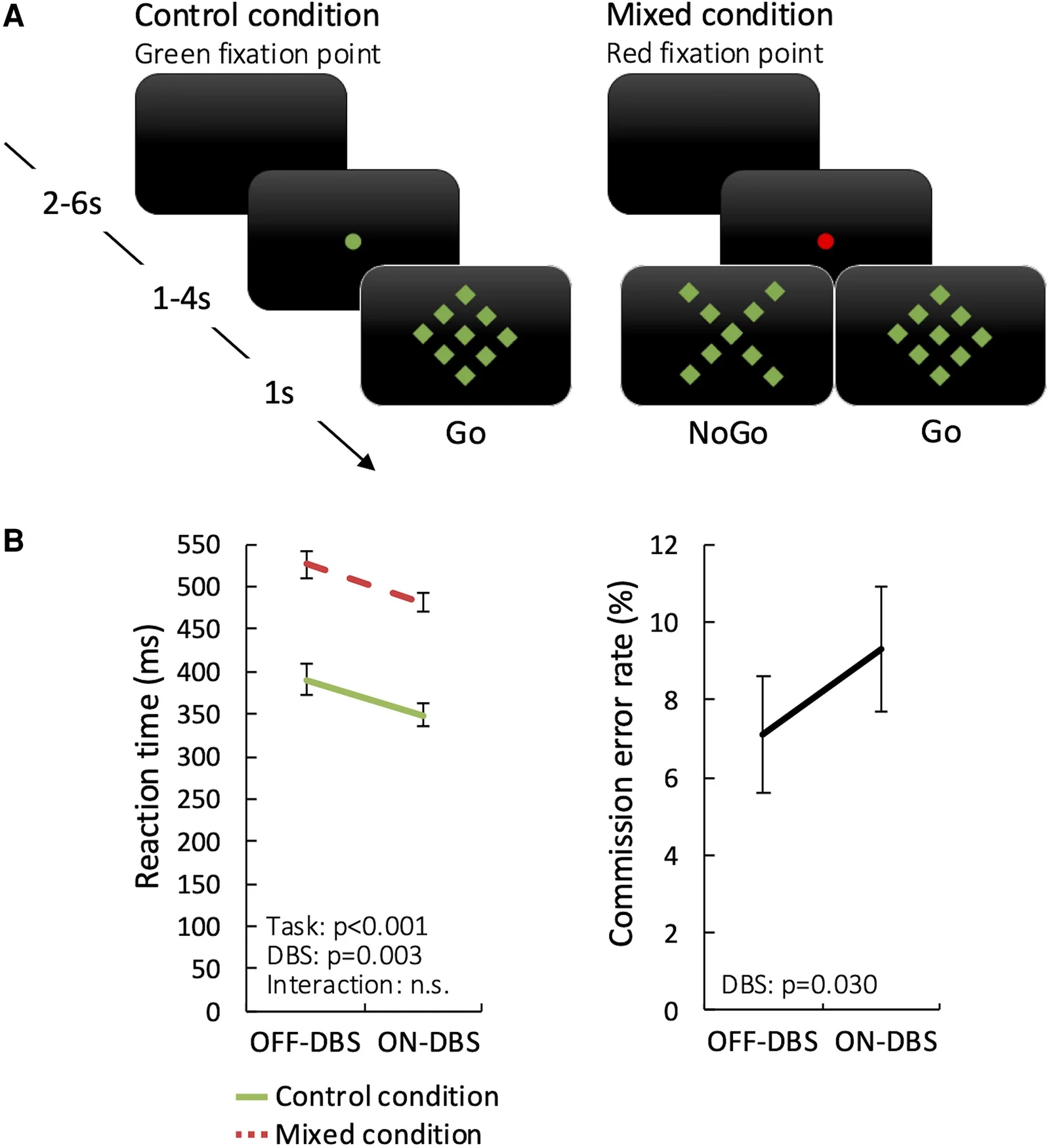
**Behavioural task and results**. (**A**) Go/NoGo task assessing automatic response inhibition. Patients were asked to respond as fast as possible to Go signals, while refraining from responding to NoGo signals. In the control condition, a green fixation point indicated that only Go signals could be presented. This fully predictable context invited participants to respond automatically to any upcoming stimulus. In the mixed condition, a red fixation point indicated that either a Go (green diamond) or a NoGo (green cross) could be presented (50% probability for each). This uncertain context invited participants to refrain from reacting automatically by setting automatic response inhibition mechanisms in order to avoid responding to NoGo signals. Indeed, automatic response inhibition is intended to allow more time to settle on the best course of action before providing long latency responses.^32,51^ The ability to provide controlled responses thus requires setting-up the appropriate level of inhibition. In contrast, enabling fast automatic responses when the environment is predictable requires anticipated and transient deactivation of the inhibitory set.^70^ In healthy subjects, this control mechanism (switching from one mode to another as a function of the context) can be assessed by comparing the mixed and control conditions of the present experiment, but has been shown to be dysfunctional in Parkinson’s disease.^4,36^ In this task, commission errors (NoGo responses) directly pinpoint impulsive responding. A lengthening in reaction time indirectly reflects the implementation of automatic response inhibition by identifying associated long latency volitional responses. (**B**) Behavioural results. Subthalamic stimulation decreased reaction time (repeated measure ANOVA, *N* = 19 patients; main effect of deep brain stimulation: *F* = 11.7, *P* = 0.003; main effect of task condition: *F* = 385.6, *P* < 0.001; interaction: *F* = 0.08, *P* = 0.78) and increased commission errors (generalized linear mixed model, *N* = 19 patients, *Z* = 2.17, *P* = 0.030), consistent with a reduction of the abnormal automatic response inhibition seen in Parkinson’s disease at the pathological state. DBS: deep brain stimulation.

This procedure is substantially different from most Go/NoGo studies which are usually based on complex designs engaging several concurrent functions (i.e. high attentional and/or working memory load;^34^). Here, to limit potential confounds, we set up simple and stable stimulus–response associations (single response), reduced the complexity of identification of the stimuli to one bit of information and used equiprobable Go and NoGo trials. Thanks to these amendments, the design requires a simple speeded reaction to a single stimulus and is known to involve prepotent automaticity (i.e. fast automatic responses that take precedence over any other potential response;^71–75^). Automatic response inhibition is precisely intended to counteract these automatic motor impulses in unpredictable environments.^51^

Participants were first trained to learn the speeded simple RT task (control condition) and ensure prepotency of the response before the recordings took place (see^32,74^ for discussion). Then, they completed two blocks of each type (mixed and control) in a counterbalanced order, for a total of 70 trials of each type. EEG was recorded throughout the task using the Biosemi ActiveTwo Mk2 system with 128 + 6 electrodes (four temporal, two at the outer canthi of the eyes), in both the ON- and OFF-DBS sessions.

### EEG data processing

The EEG analyses aimed to (i) test whether DBS modulated automatic response inhibition, by assessing a source-space event-related potential associated with this process, the DMF170^51^ and (ii) test to which extent automatic response inhibition (rather than simple motor processes) accounted for changes in movement initiation latency.

Indeed, we expected that DBS effects on automatic response inhibition would not easily be parsed from effects on motor processes based on behaviour only. The behavioural task is designed such that automatic response inhibition would only be set up in the mixed condition, while automatic responding would take place in the control condition. However, previous work suggested that PD patients have difficulties in setting an appropriate level of automatic inhibition according to the context, resulting in exaggerated inhibition over voluntary movements even when the situation does not require action restraint (e.g. in the control condition of our task;^9,35^). Hence, STN-DBS effects on automatic response inhibition are not easily identified by a specific effect on movement initiation latency (i.e. RT) in the mixed condition. Instead, a reduction of movement initiation latency is also expected in the control condition (as reported in^9,15^). To test to which extent automatic response inhibition accounts for movement initiation latency, we assessed the correlation between Go RT and the amplitude of the DMF170.^51^

The analysis of the DMF170 event-related potential followed previously published methods.^51^ Briefly, we used the DBSFILT toolbox^76^ to filter out the DBS artefacts and the FASTER toolbox^77^ to pre-process the data. Finally, to identify the source of the DMF-170, group blind source separation (gBSS) was applied following a previously validated pipeline using the UWSOBI algorithm.^51,78-80^ Consistent with the standard methodological approach for event-related potential identification,^81^ the DMF-170 was identified based on its topography, latency and functional characteristics, with no other nearby or similar candidate sources satisfying these criteria. DMF170 amplitude was extracted at peak latency for each subject and condition. Full details on decomposition parameters, convergence criteria, artefact handling and component selection are provided in Supplementary Material 2.

Of note, the EEG analyses focused on the Go trials only. Since our model assumes that automatic response inhibition applies in reaction to the detection of any stimulus, even before its full identification, in uncertain contexts, the associated electrophysiological marker is observed in both Go and NoGo trials in the mixed condition.^32,51^ This means that the electrophysiological activity of interest cannot be revealed by comparing NoGo to Go trials.^32,51^ Hence, the only possible comparison allowing identification of automatic response inhibition mechanisms is a comparison across contexts with different inhibitory requirements, i.e. the Go mixed versus Go control contrast.^82^

### Statistical analyses

RT were analysed using a two DBS states (ON, OFF) by two task conditions (Go mixed, control) repeated measure ANOVA (with the data meeting assumptions for parametric testing). A two-tailed Wilcoxon signed-rank test was used for UPDRS-III scores (ON versus OFF-DBS) due to their ordinal nature and potential deviations from normality. Commission errors (NoGo responses) were analysed using a generalized linear mixed model, which accounted for the underlying Bernoulli distribution, with subject entered as a random factor and DBS state as a fixed factor (R package lme4).

Mean DMF170 amplitude was first analysed with a paired-sample *t*-test to test for the main effect of DBS (all Go trials, mixed and control conditions pooled together, with the data meeting assumptions for parametric testing). Then, a linear mixed model was fitted on the single-trial data to assess the relationship between DMF170 amplitude and RT, accounting for the hierarchical nature of the data (i.e. multiple observations per subject). The model included DMF170 amplitude as the variable to explain, subject as a random effect and log-transformed RT as well as DBS state (ON, OFF) and task condition (Go mixed, control) as fixed effects. On average, 63.0 ± 4.5 trials per patient and condition were analysed (total *N* = 4789).

## Results

### Behavioural results

DBS improved UPDRS-III scores (ON- versus OFF-DBS, *W* = 190, *P* < 0.001; mean improvement: 60 ± 15.7%, range: 17–83%). RT was shorter in the ON- than in the OFF-DBS state (main effect of DBS, *F* = 11.7, *P* = 0.003), and shorter in the Go control than in the Go mixed condition (main effect of task condition, *F* = 385.6, *P* < 0.001; Fig. 2B). No significant interaction effect was found (*F* = 0.08, *P* = 0.78). Patients made more commission errors under DBS (9.3 ± 6.9 versus 7.1 ± 6.6%, β = 0.31, *Z* = 2.17, *P* = 0.030; Fig. 2B). Of note, interindividual variability was present, which justified studying the relationship between the magnitude of the behavioural effects and structural connectivity of the stimulation volumes. Specifically, 13 patients showed decreased RT under DBS in the control condition (6 showing a small increase), 15 showed decreased RT under DBS in the mixed condition (4 showing a small increase) and 13 showed increased commission errors under DBS (5 showing decreased errors, 1 with no change). Supplementary material 3 displays the individual data.

### Structural connectivity

Both the increase in commission error rate and the decrease in RT were associated with stimulation volume overlap with fibres connecting the supplementary motor complex to the STN, but also to a smaller extent, of fibres from the surrounding cortical areas (Fig. 3A). The ‘fiber score’ (representing the sum of the fibre values traversing a patient’s stimulation volumes) significantly correlated with the change in behaviour (commissions: *r*(13) = 0.73, *P* = 0.002; RT: *r*(13) = 0.76, *P* = 0.002). While this association remained robust under leave-one-out cross validation for commissions (*r*(13) = 0.62, *P* = 0.012), it did not for RT (*P* > 0.05).

**Figure 3:**
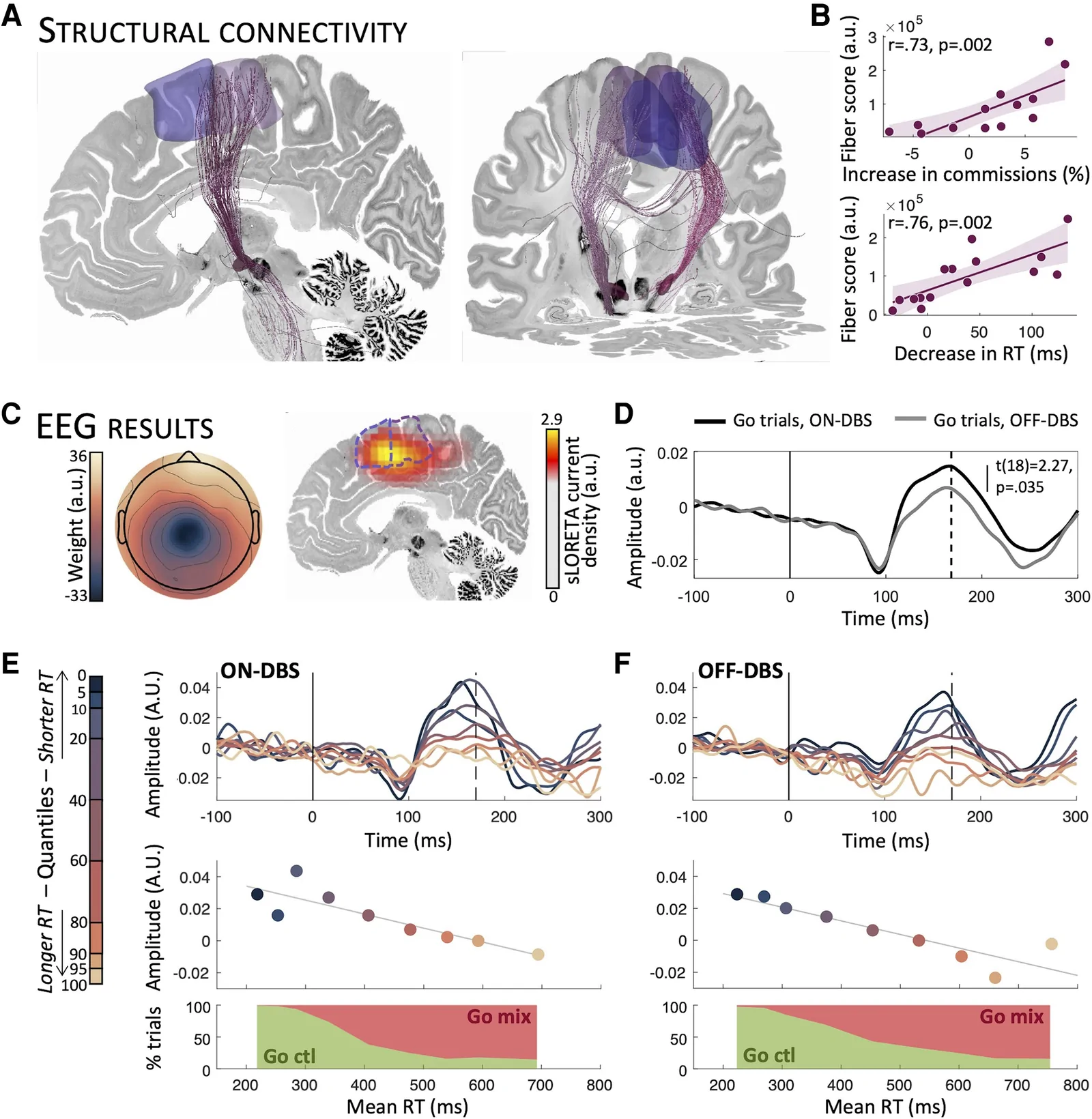
**Neural correlates of deep brain stimulation effects on movement initiation and restraint**. (**A, B**) We identified the structural connections whose stimulation accounted for the behavioural effects of stimulation. Both the increase in commission error rate and the decrease in reaction time were associated with the stimulation of fibres connecting the subthalamic nucleus to the supplementary motor complex (Pearson correlations, *N* = 15 patients, each datapoint representing an independent measure from a single patient; panel B; supplementary motor area and pre-supplementary motor area shown in purple, derived from the AAL3 atlas;^69^), but also to a smaller extent, to the surrounding areas. The fibres associated with increased commissions are shown in panel A. **(C, D)** Subthalamic stimulation modulated the DMF170, an electrophysiological marker of automatic response inhibition, which supports action restraint (paired *t*-test, *N* = 19 patients; panel D showing the DMF170 waveform). Large DMF170 amplitudes have been shown to be associated with fast uninhibited responses and are mostly observed when the context does not require action restraint. The attenuation of this motor potential, which may go as far as its complete suppression, is a direct outcome of concurrent automatic inhibition mechanisms, as previously shown.^51^ Consistent with this work,^51^ the DMF170 potential was expressed by a dorsomedial frontal component (or ‘source’; scalp map projection is shown as a topographical plot in panel C) which was localized in the supplementary motor complex, as shown by the current density map (panel C). (**E, F**) A significant correlation was found between reaction time and DMF170 amplitude when fitting a linear mixed model on single-trial data (*N* = 19 patients; β = −0.10, t = −4.87, *P* < 0.001). The largest DMF170 were found for the fastest trials (which mostly belonged to the control condition), while the smallest or no potential at all were found for the slowest trials (which mostly belong to the mixed condition). Here, for easier visualization, we represent this relationship separately for the ON- (panel E) and OFF-DBS (panel F) conditions, after partitioning all the trials in nine bins according to reaction time quantiles (each datapoint in the correlation plots representing a quantile). For both panel E (ON-DBS) and F (OFF-DBS), the top panel shows the DMF-170 waveform across reaction time quantile bins, the middle panel the correlation between reaction time and DMF170 amplitude across bins, and the bottom panel the proportion of Go trials from the control versus mixed condition across bins. In accordance with the interpretation suggesting that inhibition attenuates the motor activity giving rise to the DMF170 potential, the reaction time reduction observed under stimulation can be associated with a decrease in automatic inhibition of response in the supplementary motor complex. a.u.: arbitrary unit; DBS: deep brain stimulation; EEG: electroencephalography; RT: reaction time.

Follow-up analyses showed that the structural connectivity strength (i.e. number of fibres) between the stimulation volumes and the SMA and pre-SMA, but not between the stimulation volumes and the IFG or primary motor cortex, nor the size of the stimulation volumes, significantly correlated with the behavioural changes. Follow-up analyses were also conducted separately for the left and right hemispheres, which did not reveal a consistent pattern suggestive of lateralization. Finally, UPDRS-III improvement was not found to be significantly correlated with structural connectivity variables (see Supplementary Material 5 for detailed results).

### EEG results

DMF170 amplitude was higher in the ON- than in the OFF-DBS state (*t*-test; *t*(18) = 2.27, *P* = 0.035; Fig. 3B) and correlated with log-transformed RT (linear mixed model fitted on the single-trial data; intercept: β = 0.049, *t* = 4.33, *P* < 0.001; RT: β = −0.10, *t* = −4.87, *P* < 0.001; DBS: β = 0.004, *t* = 1.52, *P* = 0.13; task condition: β = 0.011, *t* = 3.94, *P* < 0.001, Fig. 3C). Importantly, the statistical model included ‘subject’ as a random effect, accounting for between-subject effects, meaning that the correlation reflected –at least in part– within-subject effects. Moreover, additional analyses confirmed that this correlation reflected actual changes in DMF170 amplitude rather than latency (see Supplementary Material 4).

## Discussion

### STN-DBS modulates automatic response inhibition

Here, we show that STN-DBS broadly reduces reaction time, both in situations in which patients must refrain from reacting (the mixed condition, in which Go and Nogo trials are unpredictably mixed within the same block) and situations in which they are encouraged to react automatically (the control condition, in which only Go trials are presented). Theoretically, this effect could just be accounted for by motor improvements.^15,16^ However, the increase in commission errors (impulsive responding, a variable that was not analysed in a previous contradictory study;^15^) that goes along with RT reduction also suggests a weakening of response inhibition. It is therefore likely that RT reduction is partly due to reduced inhibitory control, including when subjects are free to react automatically. Accordingly, the effect of STN-DBS can be interpreted as a partial release of the inhibitory control state into which patients are locked at the pathological state, and which substantially affects movement initiation delays even when the situation does not require restraint.^4,35,36^ Analysing an electrophysiological marker of inhibition rather than behavioural variables only, as recommended by other authors,^46,83^ reinforces this interpretation. STN-DBS was found to modulate DMF170 amplitude, and DMF170 amplitude was found to directly account for RT. Indeed, larger amplitude (i.e. weaker inhibition counteracting the motor potential) was associated with shorter RT (Fig. 3).

### STN-DBS effects on automatic response inhibition are supported by the SMC-STN loop

Evidence is still sparse regarding the network supporting automatic response inhibition. The SMC is thought to play a pivotal role, either by setting-up a self-inhibitory circuitry that gates information within the SMC itself^51^ or by relaying the braking signal to the STN via the hyperdirect SMC-STN pathway.^33,34^ However, direct evidence is still missing because of technical difficulties in acquiring reliable signal due to the small size, deep location and anatomical complexity of the STN.^84^ While recent studies using invasive electrophysiology in PD patients have demonstrated the role of the STN in the regulation of inhibition in uncertain contexts in a similar task,^85^ the functional coupling between the SMC and the STN remains difficult to access. For instance, a recent fMRI connectivity study did not provide evidence of coupling between the SMC and STN during automatic response inhibition in healthy subjects.^33^ While the present results cannot firmly distinguish between the two above-mentioned hypotheses, they support the involvement of the STN-SMC loop in the modulation of automatic response inhibition by STN-DBS. In fact, DBS modulated SMC activity associated with inhibition (i.e. the DMF170), and stimulation volume overlap with fibres connecting the STN to the SMC was associated with greater disruption of inhibitory control (as observed on commission errors) and greater improvement of the ability to initiate movements (as observed on RT; Fig. 3A). Of note, the association with commission errors remained robust under cross-validation, whereas the corresponding effect for reaction times did not, and should therefore be interpreted with greater caution.

The present study further supports the idea that stopping of ongoing movements and automatic inhibition of prepotent or automatic responses rely on partly separate networks. Of particular interest in the present dataset is the fact that the IFG was not identified as a key part of the network supporting automatic response inhibition while it is a key component of the stopping network.^29,30,39,42,43,47,86^ Indeed, no source explaining at least 1% of variance was found in the IFG (Supplementary Material 2), and the structural connectivity strength between the STN and the IFG did not account for the observed changes in behaviour (Supplementary Material 5). Functionally, the STN is likely able to integrate various signals from different hyperdirect connections^18^ in order to drive inhibition in the motor circuits when necessary, whether the instruction for global suppression comes from the identification of a stop signal or unexpected event (through the IFG-STN pathway;^30^) or from any automatic motor impulse when action restraint is required (through the SMC-STN pathway;^32^). However, it remains unclear whether the SMC region involved in automatic response inhibition is the same hub as for the other more documented forms of inhibition.^87-89^ Although the source of the DMF170 potential seems to originate from the pre-SMA rather than the SMA-proper (Fig. 3 and ^51^), the method lacks spatial resolution to precisely identify the involved cortical site.

### Mechanisms of action

The mechanisms of action of STN-DBS on the motor aspect of the disease are well documented. DBS likely exerts motor benefit by suppressing the propagation of pathological beta band activity in the cortico-subthalamo-cortical motor loop.^90-93^ STN-DBS is known to induce complex modulations of the surrounding cellular elements, including cell bodies but also axons,^94,95^ which can be activated both in the orthodromic and antidromic directions.^24,47,96-98^. In particular, the antidromic activation of the hyperdirect pathway has emerged as a potential major determinant of STN-DBS effects.^23-25,39,99,100^ Yet, the mechanisms by which DBS would affect the functional networks supporting executive functions, including automatic response inhibition, are still obscure and cannot be inferred based on the present study. It is tempting to interpret our structural connectivity findings as consistent with antidromic activation of the SMC-STN hyperdirect pathway. Greater inclusion of the fibres linking the STN to the upstream SMA/pre-SMA complex in the stimulation volumes, which may correspond to greater potential for cortical invasion by antidromic spikes, corresponded to larger effects (Fig. 3). Still, while consistent with the fundamental role of the hyperdirect pathway in motor function and dysfunction,^101^ this interpretation remains speculative in the absence of direct, pathway-specific experimental manipulations, and it is unclear how high frequency stimulation may modulate the lower-frequency activity patterns supporting the motor and executive functions of the SMC.^24,102-104^

### Limitations

This study is not exempt from limitations. These include the small sample size (albeit typical for DBS experiments;^4,9,15,39,48^), the inherent imprecision in electrode localizations introduced by the co-registration and normalization of the images, despite the application of a widely used, validated neuroimaging pipeline,^54,105-107^ and the use of gBSS.^78,79^ While gBSS is best suited to specifically extract our marker of automatic response inhibition in the SMC,^82^ it only allows analysing group-level, average effect of experimental conditions but does not offer reliable estimates of the magnitude of this effect for a given individual patient as compared to others. Hence, its relationship with structural connectivity could not be assessed. Moreover, connectivity analyses were based on a normative connectome and therefore do not capture interindividual anatomical variability, although such approaches have been shown to explain comparable variance in clinical outcomes to patient-specific connectivity at the group level.^108^ Finally, albeit consistent with the standard methodological approach for event-related potential identification,^81^ the DMF-170 was identified based on its topography, latency and functional characteristics, rather than based on quantitative metrics.

## Conclusion

Here, we show that STN-DBS reduces action restraint and facilitates movement initiation by modulating the network supporting automatic response inhibition. From a clinical perspective, this means that stimulation settings that maximize the improvement of movement initiation may also promote impulsive behaviour.^5-7,9,40^ Of note, although the increase in commission error rate under DBS is relatively small on average, some patients experienced stronger increases. The clinical relevance of these changes warrants further investigation. With impulsivity taking many forms in PD, impaired inhibitory control under STN-DBS may contribute, in some patients, to subtle behavioural changes in daily life,^109^ or even to clinically significant impulsivity in the form of impulse control disorders.^40,110^ Moreover, the degree of dissociation between the networks and mechanisms supporting DBS-induced benefits on overall motor function—beyond movement initiation—and development of impulsive behaviour remains an open issue.^5,6,39,40^ Indeed, we did not observe significant correlations between clinical (UPDRS-III) and behavioural or connectivity measures. This may reflect differences in sensitivity and the fact that these metrics capture partially distinct aspects of DBS effects. Future studies with larger samples and symptom-specific analyses will be needed to clarify these relationships. A better understanding of the involved networks may offer opportunities to better balance between the various therapeutic benefits and adverse effects of stimulation as a function of network-specific neuromodulatory effects.^14,111^ Finally, by identifying a symptom/side-effect pathway related to the ability to initiate movement, our study contributes to the building of a library of dysfunctional circuits that can be targeted for neuromodulation, recently termed the ‘dysfunctome’.^112^

## Supplementary Material

fcag325_Supplementary_Data

## Data Availability

The data that supports the findings of this study is available on request from the corresponding author. The data are not publicly available due to privacy or ethical restrictions. Software tools used for this study are publicly available through GitHub repositories maintained by the respective software developers (https://github.com/guillaumelio/DBSFILT; https://github.com/sccn/eeglab; https://github.com/netstim/leaddbs).
