# Supplementary material for: Hyperdirect projections from the supplementary motor area mediate subthalamic stimulation effects on movement initiation and inhibition in Parkinson’s disease
Source: Brain Commun. 2026 Aug 28;8(5):fcag325. doi: 10.1093/braincomms/fcag325 (PMC13644363; doi:10.1093/braincomms/fcag325)
Supplement: Supplementary file 1 [file fcag325_supplementary_data.pdf]

Supplementary material

Meyer et al. – SMA-STN hyperdirect projections mediate subthalamic stimulation effects on movement initiation and inhibition in Parkinson's disease

Contents

Supplementary material 1:	Patients' characteristics	p. 2
Supplementary material 2:	EEG data processing	p. 4
Supplementary material 3:	Interindividual variability	p. 7
Supplementary material 4:	Relationship between EEG activity and movement onset	p. 8
Supplementary material 5:	Supplementary structural connectivity analyses	p. 10

Supplementary material 1: Patients' characteristics

	Age	Sex	Laterality	LEDD	DD	TS	UPDRS-III ON	UPDRS-III OFF	Right stimulation parameters	Left stimulation parameters
1	58	M	0,83	475	8	4	10	37	7-/3,6V/60µs/130Hz	3-/2,7V/60µs/130Hz
2	49	M	0,83	160	15	25	6	18	6-/3,3V/90µs/130Hz	1-/3,3V/90µs/130Hz
3	64	F	0,83	770	23	77	20	24	5-/ 2,8V/60µs/130Hz	2-/ 3,3V/60µs/130Hz
4	67	M	0,50	560	10	41	4	23	6-/ 2,4V/90µs/160Hz	2-/ 2,6V/90µs/160Hz
5	43	M	0,67	600	8	23	23	35	6-/ 2,9V/60µs/130Hz	3-/ 3,7V/60µs/130Hz
6	64	F	1,00	610	26	24	11	24	7-/ 2,9V/90µs/160Hz	3-/ 3,4V/90µs/160Hz
7	61	M	1,00	450	23	45	23	48	6-,7-/ 3V/90µs/130Hz	2-/ 3,2V/60µs/130Hz
8	50	F	0,83	750	10	3	4	19	6-/ 2,8V/60µs/130Hz	3-/ 3V/60µs/130Hz
9	64	F	0,92	262,5	24	48	26	60	5-/3,3V/90µs/130Hz	1-/3,4V/60µs/130Hz
10	68	F	0,91	575	7	18	5	16	6-/2,6V/60µs/130Hz	2-/2,7V/60µs/130Hz
11	69	M	0,75	450	13	59	12	38	7-/2,6V/60µs/160Hz	3-/3,4V/60µs/160Hz
12	59	M	0,83	200	9	3	13	33	9-/ 3V/60µs/130Hz	1-/ 2,8V/60µs/130Hz
13	70	M	0,83	710	13	63	25	55	6-/ 3,2V/60µs/130Hz	2-/ 3,1V/60µs/130Hz
15	60	M	0,58	940	12	51	4	11	6-/ 3,4V/60µs/130Hz	2-/ 3,9V/60µs/130Hz
16	55	M	1,00	0	13	21	16	48	6-/ 3V/60µs/160Hz	2-/ 3,3V/60µs/160Hz
17	65	M	1,00	800	11	29	8	19	5-/ 4,2V/60µs/70Hz	2-/ 4V/60µs/70Hz

18	51	M	0,83	1030	8	43	6	30	6-/ 3,7V/90µs/130Hz	2-/ 2V/60µs/130Hz
19	58	M	1,00	225	11	35	18	44	5-/ 3,2V/60µs/130Hz	1-/ 3V/60µs/130Hz
20	59	M	0,70	825	10	54	29	59	5-/ 3,5V/60µs/130Hz	1-/ 3,4V/60µs/130Hz
	59,7	***5F***		***547,0***	***13,4***	***35,1***	***13,8***	***33,7***		
	7,2			***274,6***	***5,9***	***20,5***	***8,2***	***14,8***		

Supplementary table 1: Patients' characteristics. LEDD: Levodopa equivalent daily dose; DD: Disease duration, years; TS: Time since surgery, months; UPDRS-III ON and OFF-DBS assessed at the time of the experiment. Laterality index refers to handedness as assessed by the Edinburgh Inventory (positive scores indicate a right preference, while negative scores reflect a left preference; no left-handed patients were included in the cohort). Stimulation parameters are reported in Medtronic notation (0 and 4 are the most distal contacts, C refers to case).

Supplementary material 2: EEG data processing

Task stimuli

All stimuli were presented on a light-emitting diode panel (LED, Ø5 mm, 8800 mcd). A central LED served as a fixation point. Go (green diamonds) and NoGo (green crosses) stimuli were composed of eight LEDs centered on it (3.44° of visual angle, stimulus duration: 50 ms).

Pre-processing

When recording ON-DBS, the EEG signal is corrupted by DBS artifacts, that may not be completely eliminated by standard low-pass filtering (1,2). Thus, we used the dedicated open-source toolbox DBSFILT (https://github.com/guillaumelio/DBSFILT/releases) to filter out the DBS artifacts. The toolbox combines low-pass, zero-phase, digital temporal filtering with frequency-domain filtering based on artifact identification procedures that allow removing and interpolating aliased frequencies (1). This procedure was applied before bandpass filtering (0.75-45 Hz) and downsampling (1024 Hz). Then, epoching and automatic epoch rejection were performed using the FASTER toolbox (3). Epochs were cut from 1500 ms before to 1000 ms after stimulus onset, for both Go and NoGo stimuli. Data was converted to average reference. Finally, individual source separation (SOBI algorithm, 4) was performed using the EEGlab toolbox (5) to reject the artifactual components (e.g., eyes movements, blinks, muscle activity). For defective channels, spherical interpolation was privileged over rejection of the corresponding artifactual components.

Group blind source separation (gBSS) and selection of the component of interest

We used blind source separation (BSS), a procedure that allows to "unmix" the overlapping brain activities of the EEG signal without the need of any a priori information about the sources or the mixing process (6,7), to track the DMF170. Indeed, this early marker is obscured by powerful concurrent (mainly visual) activity in the original signal (8). More precisely, we used group blind source separation (gBSS) since it can readily extract components that are commonly expressed at the group level (9–11).

The entire analysis pipeline followed published methods (8–10). gBSS was applied on aggregated data, including all trials obtained from the patients (i.e., Go and NoGo trials), using the UWSOBI algorithm (4). Indeed, algorithms based on second order statistics have been shown to be more robust than those based on higher order statistics with regard to anatomo-functional inter-individual variability, and are thus more suitable for gBSS (9,10). Two hundred lagged-covariance matrices with time delays ranging from 0/1024s to 200/1024s were calculated for each epoch. The lagged-covariance matrices were averaged across the patient's epochs and then across all patients and conditions, so that 200 lagged covariance matrices were approximately joint-diagonalized with the UWEDGE algorithm using its default stopping criteria (12). This led to the identification of 134 components (ICs). The sLORETA software (13) was used to localize the components based on a head model obtained by applying the boundary element method to the MNI152 template (solution space restricted to cortical grey matter, 6239 voxels at 5mm resolution, Tikhonov regularization optimized for a signal/noise ratio of 100; 14). The components' activity time-courses were reconstructed by applying the group BSS filter (i.e., weights) to individual subjects' data, filtered under 30Hz, and baseline-corrected (baseline: [-200, 0ms]).

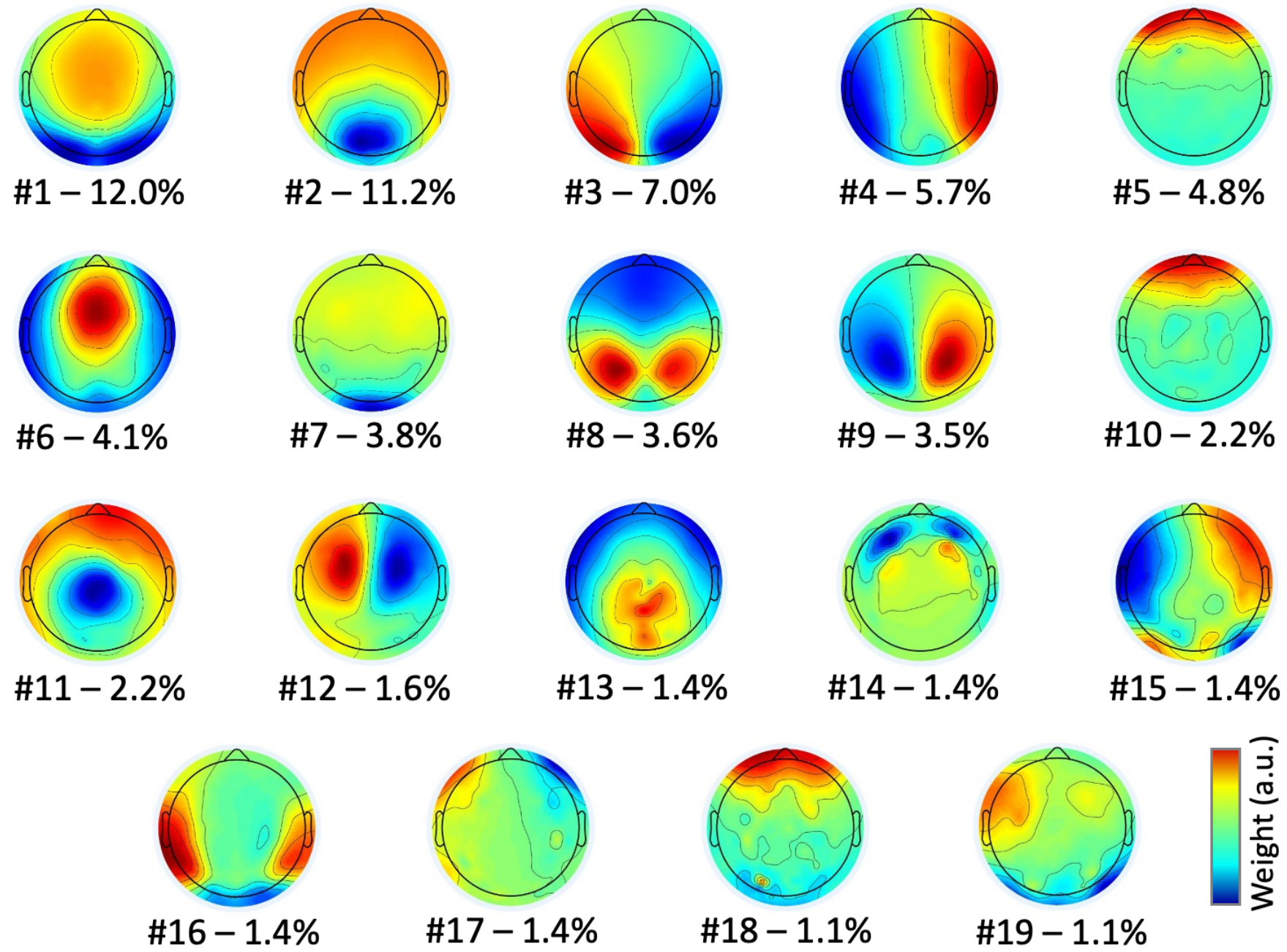

Supplementary Figure 1: Topographies of the group blind source separation components explaining more than 1% of variance (N=19 patients). Color scales indicate component weights at each electrode in arbitrary units (a.u.).

We identified a component consistent with the features of the DMF170 (8) among those explaining more than one percent of variance (N=19 out of 134; Supplementary Fig. 1). Following standard practice in the field (15), we identified this component based on its topography, peak latency, and functional characteristics. Specifically, we first looked for components characterized by a topography consistent with a medial frontal source and a sLORETA localization probability map encompassing the supplementary motor complex (two components, IC#11 and #12). Second, we searched for the one whose activity reproduced the temporal characteristics of the DMF170, that is, being locked to stimulus presentation and with a peak latency around 168 ms (8; Supplementary Fig. 2). One component was clearly identified (IC#11). Indeed, visual inspection of the grand average waveforms revealed that activity consistent with the DMF170 (with remarkable correspondence of peak latency as compared to the original report) was present in IC#11, but not IC#12 (Supplementary figure 2). Identification of the DMF170 was further confirmed by its functional characteristics, also described in the original study (8), that is, a correlation between amplitude and reaction time and corresponding difference in amplitude between the control and mixed task conditions (see Results).

Thus, mean DMF170 amplitude was extracted from IC#11. Mean amplitude was extracted at 168ms (i.e. the peak latency of the DMF170 as previously identified in 8) for each patient and DBS state. Finally, for single-trial analyses, IC#11 activity amplitude at the same latency was extracted for each trial.

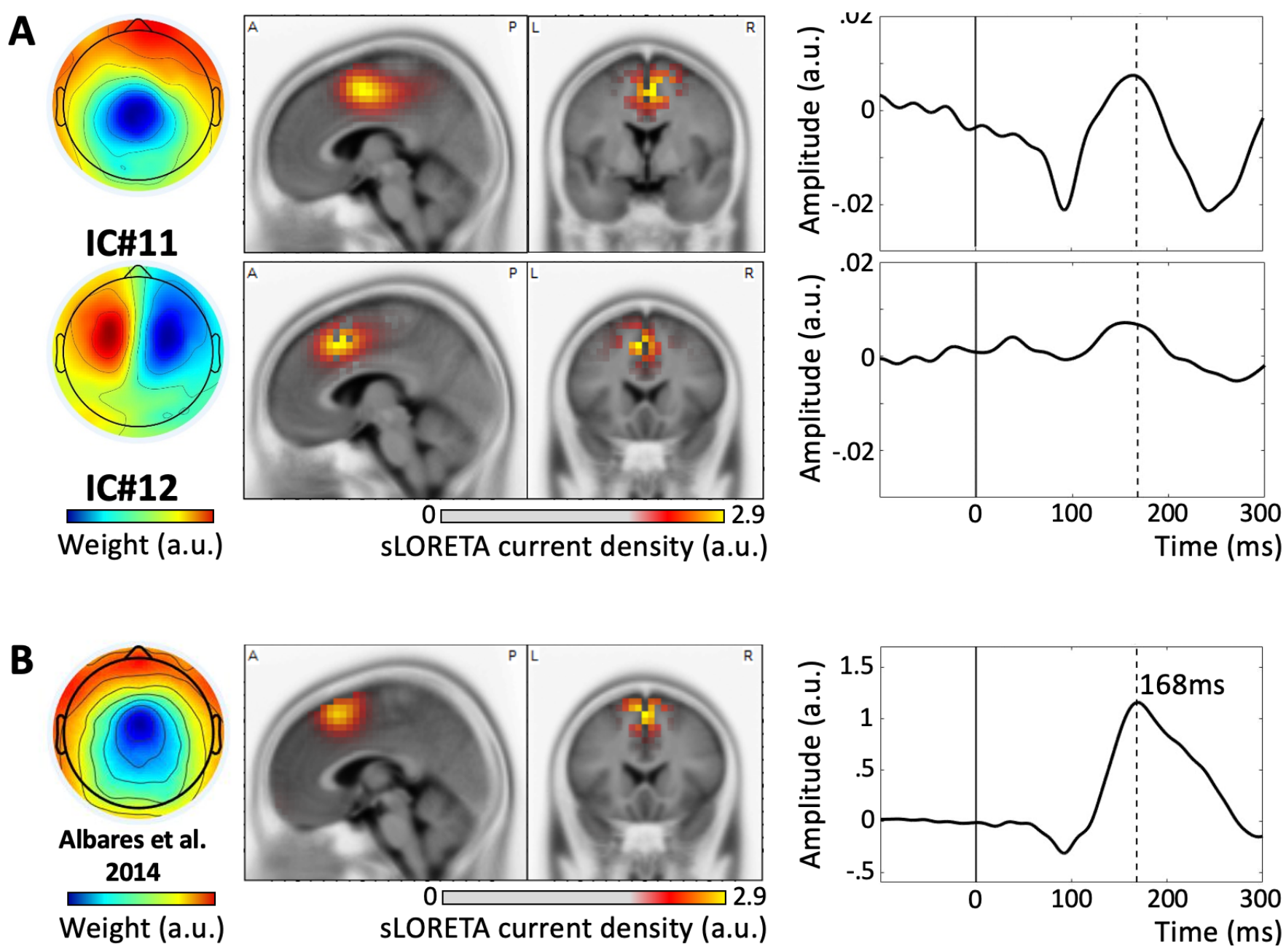

Supplementary figure 2: Identification of the DMF170. (A) Characteristics of the two medial frontal components (IC#11 and IC#12) identified in the present study (N=19 patients). Topography, localization and event-related potential of IC#11 resembled those of the DMF170 component previously identified in healthy subjects (8). (B). Illustrations for panel B are adapted from (8). This previous study included 20 healthy subjects. Note that in the original paper (5), and unlike in the present figure, the DMF170 event-related potential was back-projected to the scalp and thus appeared as a negative-going deflection. This operation was only applied to recover the original unit –μV– and to facilitate the comparison with other scalp-level event-related potentials. Given that comparison with scalp-level potentials was not required in the present study, source activity was directly analyzed, rather than back-projected to a particular scalp electrode as in the original report. Of note, in blind source separation, the polarity of extracted components is inherently arbitrary (i.e., the results of the decomposition can be equivalently represented with inverted sign). Importantly, the polarity does not affect amplitude-based or statistical analyses, which depend on the magnitude of the neural response. Therefore, the polarity inversion does not impact comparability with the original scalp-level findings. a.u.: arbitrary unit; IC: independent component.

Consistency test

Finally, we ran a consistency test (as reported in 15,16) to ensure that the component of interest was reasonably replicable across patients. This aimed to rule out the possibility of a poor representation of the component (and hence of the corresponding activity of interest) in some of the patients. This test consists in separating the cohort in two subgroups that are the most similar regarding their clinical characteristics and behavior (here we used age, LEDD, disease duration, UPDRS-III ON and OFF, RTs ON and OFF and commission error rates ON and OFF), and then computing a quality index Q_i based on the spatial correlations within triplets of ICs that are deemed the most similar among the entire group and the subgroups. A complete description of the test implementation is available elsewhere (17). According to this test, the DMF170 component was considered reasonably replicable across subjects (IC#11: Q_i=61%, p=.004).

Supplementary material 3: Interindividual variability

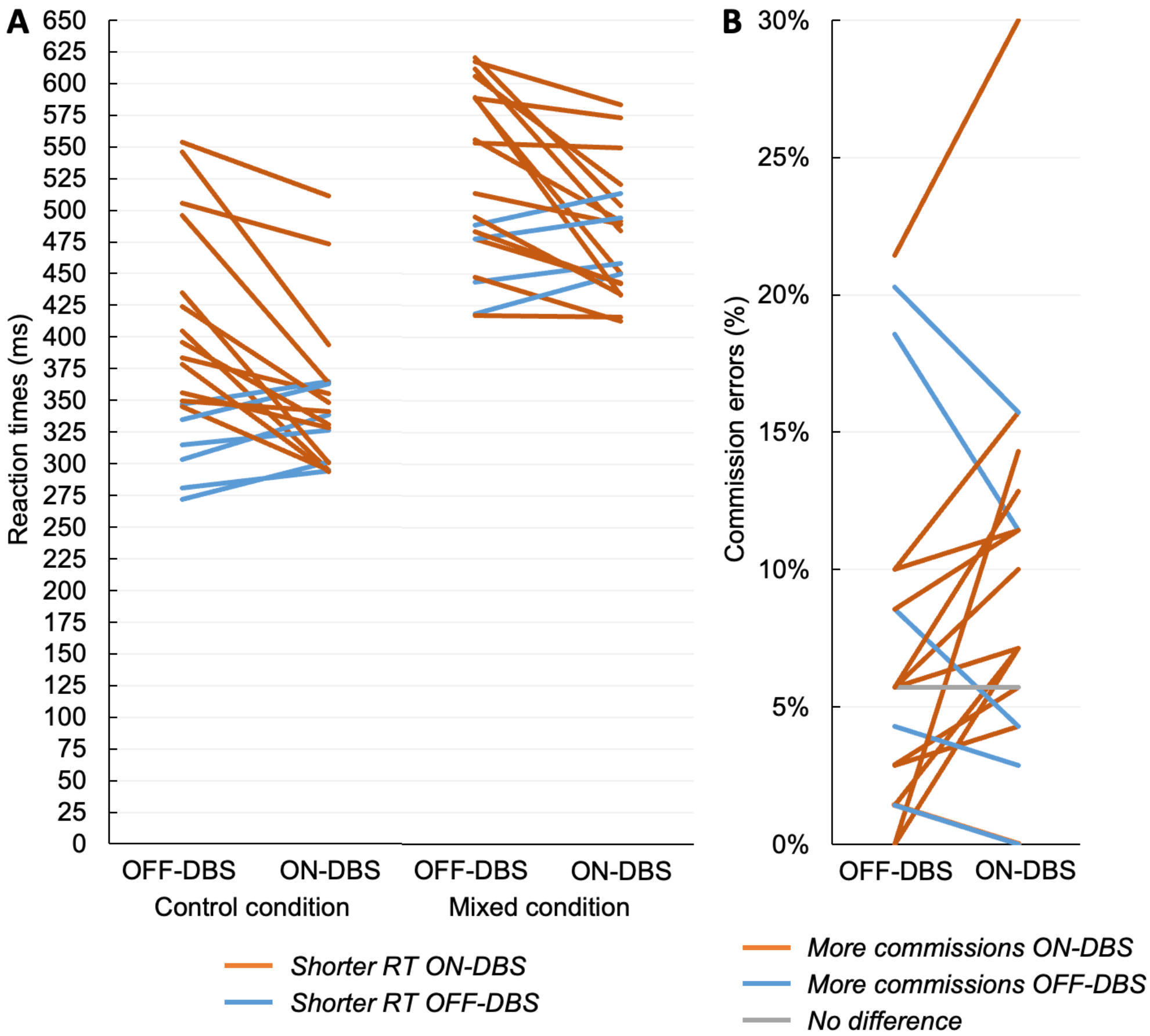

Supplementary figure 3: Individual data for reaction times and commission error rates (each line representing one patient, N=19). DBS: deep brain stimulation; RT: reaction time.

Supplementary material 4: Relationship between EEG activity and movement onset

The control of movement-related changes is an inherent problem when testing for correlations with RT in neurophysiology. Indeed, the correlation between RT and DMF170 amplitude may be explained by a relationship of the DM170 to movement onset instead of stimulus occurrence (i.e., the DMF170 may be a movement-locked rather than a stimulus-locked activity). The stronger variability would hence explain the lower amplitude of this ERP with longer RTs. To investigate this, we plotted IC#11 activity across trials, pooling Go trials from all subjects. For visualization, a trial moving average smoothing has been applied (window length: 200 trials). We then tested the correlation between ERP peak latency and RT (see below). To this aim, Go trials (Go_mix and Go_ctl) from all subjects were pooled together and partitioned in nine bins according to RT distribution percentiles. ERP peak latency and mean RT were computed for each bin, and the correlation was evaluated using the Spearman correlation coefficient. Finally, we extracted the response-locked activity of IC#11 (epoch limits: [-1500, 200ms] relative to RT, baseline: [-1500, -1300ms]). Note that for this control response-locked analysis, 30 trials OFF-DBS (out of 2423) and 11 trials ON-DBS (out of 2367) with RTs above 800ms were excluded, to allow the re-epoching to be done on the existing epochs ([-1500, 1000ms] relative to stimulus occurrence) rather than on the continuous data.

When plotting IC#11 activity across trials, the first ERP observable component locked to stimulus presentation is the DMF170. A second ERP component peaking close to RT was observed (Supplementary figure 4A). Its latency was significantly correlated with RT (ON: ρ=.98, p<.001; OFF: ρ=.99, p<.001; Supplementary figure 4B). Aligning the data on movement onset (RT) clearly revealed this response-locked activity (Supplementary figure 5).

Overall, these results support the original interpretation of the DMF170 component (Albares et al 2014) as a marker of automatic motor activation consecutive to stimulus presentation, whose attenuation is a direct outcome of inhibition. On the other hand, we suggest that the second ERP component locked to the motor response likely reflects the motor activity related to the execution of long latency voluntary responses.

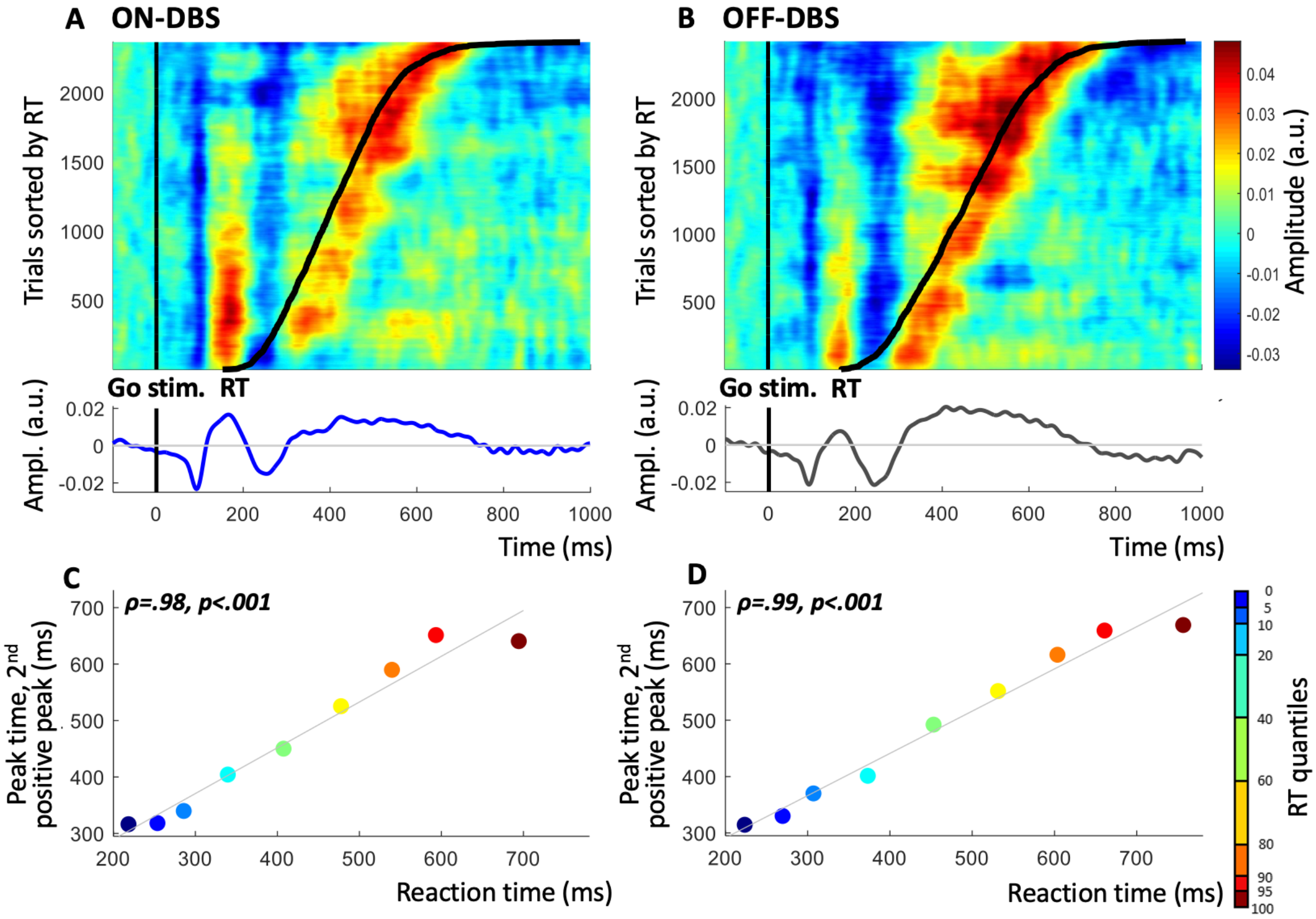

Supplementary figure 4: Relationship between IC#11 activity and reaction time. (A,B) Event-related activity of IC#11 displayed across all Go trials (Go_mix and Go_ctl) sorted by reaction time, in the ON- and (A) OFF-DBS (B) conditions. (C,D) The second event-related component's peak time significantly correlates with reaction time at the group level, both in the ON- (C) and in the OFF-DBS (D) conditions (Spearman correlation across nine reaction time quantiles, each represented by one datapoint in the correlation plot; ON: ρ=.98, p<.001; OFF: ρ=.99, p<.001). a.u.: arbitrary unit; ampl.: amplitude; DBS: deep brain stimulation; RT: reaction time; stim.: stimulus.

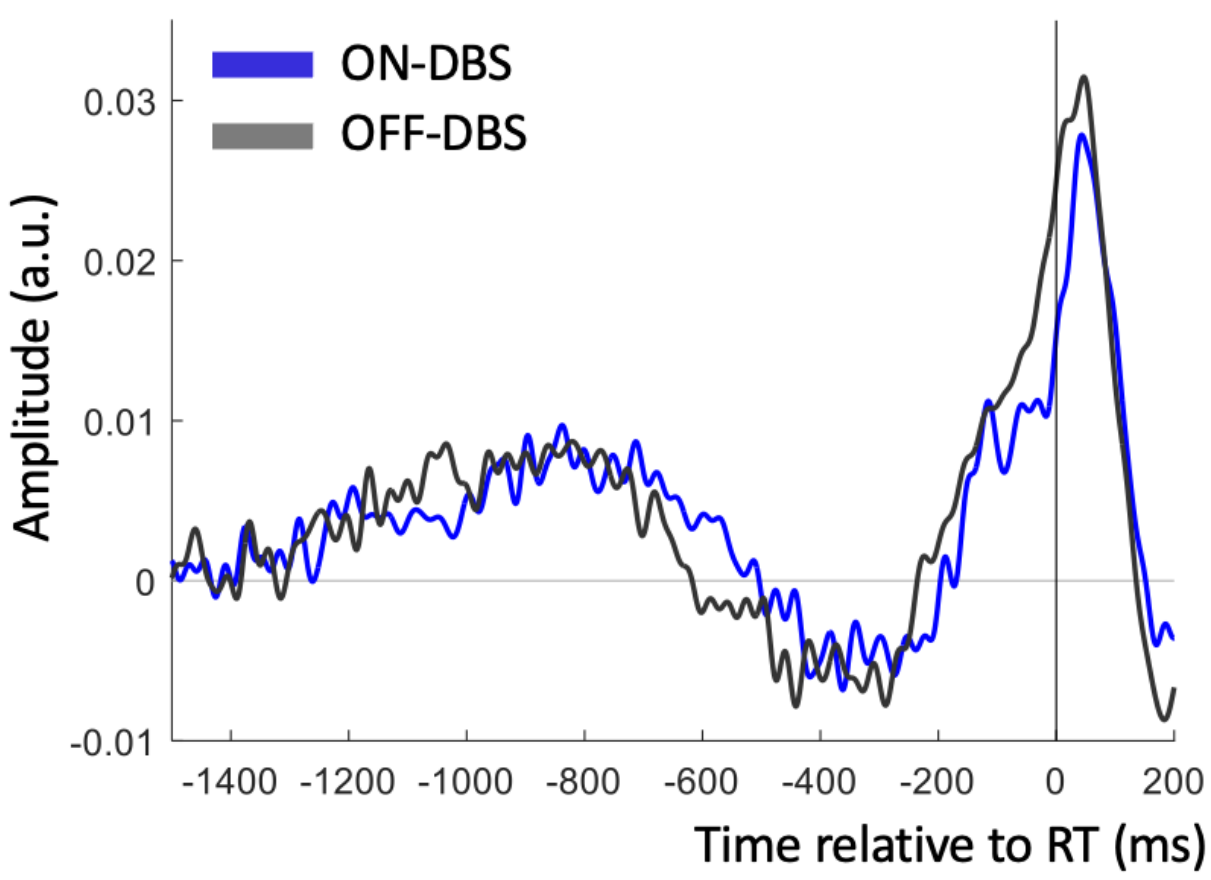

Supplementary figure 5: Response-locked activity for IC#11 (N=19 patients).

Supplementary material 5: Supplementary structural connectivity analyses

Supplementary table 2: Correlations between change in behavior in the Go/NoGo task and strength of structural connectivity between stimulation volumes (VTAs) and ROIs. Pearson coefficients are reported only when assumptions are met. All tests were conducted on N=15 patients.

	Change in commission error rate (%)	Change in mean reaction time (OFF-ON, ms)
Correlations with connectivity to SMC, M1 and IFG		
Structural connectivity strength, VTAs-bilateral pre-SMA	r=.58, p=.023<br>ρ=.44, p=.099	r=.67, p=.007<br>ρ=.57, p=.029
Structural connectivity strength, VTAs-bilateral SMA	r=.52, p=.048<br>ρ=.49, p=.065	r=.49, p=.061<br>ρ=.48, p=.073
Structural connectivity strength, VTAs-bilateral M1	-<br>ρ=.35, p=.20	r=-.03, p=.91<br>ρ=.06, p=.84
Structural connectivity strength, VTAs-bilateral IFG	r=.41, p=.13<br>ρ=.40, p=.14	r=.35, p=.20<br>ρ=.49, p=.06
Lateralization effects in the correlations with connectivity to SMC		
Structural connectivity strength, VTAs-left pre-SMA	r=.48, p=.069<br>ρ=.24, p=.38	r=.45, p=.089<br>ρ=.47, p=.076
Structural connectivity strength, VTAs-right pre-SMA	r=.57, p=.025<br>ρ=.44, p=.10	r=.72, p=.002<br>ρ=.63, p=.014
Structural connectivity strength, VTAs-left SMA	r=.52, p=.045<br>ρ=.55, p=.032	r=.41, p=.13<br>ρ=.31, p=.25
Structural connectivity strength, VTAs-right SMA	r=.33, p=.23<br>ρ=.31, p=.26	r=.40, p=.13<br>ρ=.47, p=.081
Correlations with total stimulation volume		
Total stimulation volume (mm^3)	r=.22, p=.42<br>ρ=.27, p=.33	r=.37, p=.17<br>ρ=.21, p=.45

Supplementary table 3: Correlation between change in clinical motor scores and strength of structural connectivity between stimulation volumes (VTAs) and SMC. All tests were conducted on N=15 patients.

	UPDRS-III improvement (%)	UPDRS-III improvement, bradykinesia subscore (%)
Structural connectivity strength, VTAs-bilateral pre-SMA	-<br>ρ=-.12, p=.66	-<br>ρ=-.02, p=.93
Structural connectivity strength, VTAs-bilateral SMA	r=.20, p=.47<br>ρ=.08, p=.77	-<br>ρ=.10, p=.72

Supplementary References

1. Lio G, Thobois S, Ballanger B, Lau B, Boulinguez P. Removing deep brain stimulation artifacts from the electroencephalogram: Issues, recommendations and an open-source toolbox. Clin Neurophysiol. 2018 Oct;129(10):2170–85. doi:10.1016/j.clinph.2018.07.023 PubMed PMID: 30144660.

2. Litvak V, Florin E, Tamás G, Groppa S, Muthuraman M. EEG and MEG primers for tracking DBS network effects. NeuroImage. 2021 Jan 1;224:117447. doi:10.1016/j.neuroimage.2020.117447

3. Nolan H, Whelan R, Reilly RB. FASTER: Fully Automated Statistical Thresholding for EEG artifact Rejection. Journal of Neuroscience Methods. 2010 Sep 30;192(1):152–62. doi:10.1016/j.jneumeth.2010.07.015

4. Belouchrani A, Abed-Meraim K, Cardoso JF, Moulines E. A blind source separation technique using second-order statistics. IEEE Transactions on Signal Processing. 1997 Feb;45(2):434–44. doi:10.1109/78.554307

5. Delorme A, Makeig S. EEGLAB: an open source toolbox for analysis of single-trial EEG dynamics including independent component analysis. J Neurosci Methods. 2004 Mar 15;134(1):9–21. doi:10.1016/j.jneumeth.2003.10.009 PubMed PMID: 15102499.

6. Onton J, Westerfield M, Townsend J, Makeig S. Imaging human EEG dynamics using independent component analysis. Neuroscience & Biobehavioral Reviews. 2006 Jan 1;30(6):808–22. doi:10.1016/j.neubiorev.2006.06.007

7. Makeig S, Westerfield M, Jung TP, Enghoff S, Townsend J, Courchesne E, et al. Dynamic brain sources of visual evoked responses. Science. 2002 Jan 25;295(5555):690–4. doi:10.1126/science.1066168 PubMed PMID: 11809976.

8. Albares M, Lio G, Criaud M, Anton JL, Desmurget M, Boulinguez P. The dorsal medial frontal cortex mediates automatic motor inhibition in uncertain contexts: evidence from combined fMRI and EEG studies. Hum Brain Mapp. 2014 Nov;35(11):5517–31. doi:10.1002/hbm.22567 PubMed PMID: 24954611.

9. Lio G, Boulinguez P. Greater robustness of second order statistics than higher order statistics algorithms to distortions of the mixing matrix in blind source separation of human EEG: Implications for single-subject and group analyses. NeuroImage. 2013 février;67:137–52. doi:10.1016/j.neuroimage.2012.11.015

10. Lio G, Boulinguez P. How Does Sensor-Space Group Blind Source Separation Face Inter-individual Neuroanatomical Variability? Insights from a Simulation Study Based on the PALS-B12 Atlas. Brain Topogr. 2018;31(1):62–75. doi:10.1007/s10548-016-0497-z PubMed PMID: 27251709.

11. Huster RJ, Raud L. A Tutorial Review on Multi-subject Decomposition of EEG. Brain Topogr. 2018;31(1):3–16. doi:10.1007/s10548-017-0603-x PubMed PMID: 29063237.

12. Tichavsky P, Yeredor A. Fast Approximate Joint Diagonalization Incorporating Weight Matrices. IEEE Transactions on Signal Processing. 2009 Mar;57(3):878–91. doi:10.1109/TSP.2008.2009271

13. Pascual-Marqui RD. Standardized low-resolution brain electromagnetic tomography (sLORETA): technical details. Methods Find Exp Clin Pharmacol. 2002;24 Suppl D:5–12. PubMed PMID: 12575463.

14. Mazziotta J, Toga A, Evans A, Fox P, Lancaster J, Zilles K, et al. A probabilistic atlas and reference system for the human brain: International Consortium for Brain Mapping (ICBM). Philos Trans R Soc Lond B Biol Sci. 2001 Aug 29;356(1412):1293–322. doi:10.1098/rstb.2001.0915 PubMed PMID: 11545704; PubMed Central PMCID: PMC1088516.

15. Luck SJ. An Introduction to the Event-Related Potential Technique. MIT Press; 2014. 417 p.

16. Congedo M, John RE, De Ridder D, Prichep L. Group independent component analysis of resting state EEG in large normative samples. Int J Psychophysiol. 2010 Nov;78(2):89–99. doi:10.1016/j.ijpsycho.2010.06.003 PubMed PMID: 20598764.

17. Spay C, Albares M, Lio G, Thobois S, Broussolle E, Lau B, et al. Clonidine modulates the activity of the subthalamic-supplementary motor loop: evidence from a pharmacological study combining DBS and EEG recordings in Parkinsonian patients. J Neurochem. 2018 Apr 20;146(3):333–47. doi:10.1111/jnc.14447 PubMed PMID: 29675956.
